# The relationship between sleep duration and physical activity level among Norwegian adolescents: a cross-sectional study

**DOI:** 10.3389/fpubh.2024.1495826

**Published:** 2024-11-28

**Authors:** Erik Grasaas, Mari Hysing, Øyvind Sandbakk

**Affiliations:** ^1^Teacher Education Unit, University in Agder, Kristiansand, Norway; ^2^Department of Psychosocial Science, University of Bergen, Bergen, Norway; ^3^School of Sport Science, UiT The Arctic University of Norway, Tromsø, Norway

**Keywords:** adolescents, school-based sample, sleep, exercise, training, health education

## Abstract

**Background:**

Failure to adhere to sleep and physical activity recommendations among adolescents constitutes a public health problem. However, the associations between sleep duration and adolescents’ physical activity levels remain less explored. The aims of this paper were twofold: (1) to describe sleep and physical activity levels among Norwegian school-based adolescents, stratified by school level and sex and (2) to explore the association between sleep and physical activity levels.

**Methods:**

Data were derived from the 2022 Norwegian Ungdata Survey, totaling 63,113 adolescents from lower (aged 13 to 16 years) and upper (aged 16 to 19 years) secondary schools. Study variables were measured using single-item questions from the Ungdata survey and collected through an electronic questionnaire administered during school hours. Logistic regressions were performed using crude analysis and adjusted for Socioeconomic status (SES) and grade level (age).

**Results:**

In lower secondary school, 57.0% of girls and 44.7% of boys reported sleeping less than the recommended 8 h, whereas in upper secondary school, the rate was 74.9% among girls and 74.3% among boys. Girls consistently reported more sleep problems, feeling more tired at school or during activities, and less physical activity than boys across school levels. Sleep duration was a significant predictor for all levels of weekly physical activity among girls across school levels, with the highest odds revealed in upper secondary school among those being active 5 times a week (*B* = 1.32; 95% CI [1.24 to 1.40]). Sleep duration was a predictor for being active 5 times a week for boys across school levels (*B* = 1.22; 95% CI [1.17 to 1.27]).

**Conclusion:**

About half of younger adolescents and three-quarters of older adolescents do not adhere to the sleep recommendation. Lower levels of physical activity were consistently reported by girls than boys. Sleep duration consistently predicted a 20 to 30% higher likelihood of being active at least 5 days a week across sex and school levels. These findings underscore the critical role of sleep duration relations to higher physical activity levels among Norwegian adolescents. Government and policymakers should encourage healthy sleep and PA habits by explicitly incorporating guidelines into the curriculum.

## 1 Introduction

Sleep and physical activity (PA) is acknowledged as two pivotal factors for health, and is essential in functions such as memory, learning, immune function, neural development, strength, cardiovascular and metabolic health (1–3). Despite the long list of benefits, a large amount of the population fails to meet the recommendations of both sleep and PA.

Adolescents are recommended to obtain 8–10 h of sleep according to the US National Sleep Foundation (4). Many adolescents do not meet the recommendations of sleep, with estimates ranging from 32 to 86% across countries (5). In Norway, Saxvig and colleagues found that 84% did not meet the recommendation of 8-h sleep in a sample of adolescents in 1st grade of higher secondary school, aged 16–17 years old, in 2019 (6). Another Norwegian population-based study estimated three out of four adolescents in higher secondary school, from 1^st^ to 3^rd^ grade, did not meet the recommendations in 2021, and that most adolescents are tired in school and having problems falling asleep at least 1–2 days a week (7). If these estimates are representative for sleep duration and sleep problems, further investigation of younger and older adolescents is needed as the results of the major changes in adolescent life including sleep related changes in relation to the Covid-19 (8, 9).

The WHO recommends at least 60 min of moderate-to-vigorous intensity PA per day for adolescents (10). Prevalences of not meeting the PA recommendations are reported across world parts to be as high as 66 to 97% among adolescents (11). Steene-Johannessen and colleagues, standardized accelerometer measures of PA across Europe and found about two-thirds of European children and adolescents, and about half of Norwegian adolescents did not adhere to PA recommendations by using objective accelerometer (12, 13). Trends from 2017 to 2021 using self-reported data revealed higher rate of not adhering to PA recommendations, ranging from 70 to 85% among Norwegian adolescents, with girls consistently reporting less PA than boys (14). A recent Norwegian research paper suggested that even higher prevalence is evident among Norwegian adolescents when specifically asking into the daily 60-min of PA requirements, with prevalences of 91% among boys and 96% among girls (15). With the high prevalence and relevance for future health, the lack of adhering to sleep and PA recommendations among adolescents constitutes to a public health challenge (16, 17).

The commonness of not meeting the sleep and PA recommendations in adolescence is worrying as it affects all parts of their everyday life, including their energy levels, wellbeing and daytime capacities (15, 18–22). Still, there is a scarcity of evidence on the associations between sleep duration and PA among Norwegian adolescents. In other populations, such as in university students a systematic review and meta-analysis revealed that moderate to vigorous PA was associated with better sleep quality, whereas PA was weakly inversely associated with sleep duration, however findings could presumably be influenced by napping behavior (23). In adults, the association between sleep duration and exercise is suggested to be of a bidirectional nature (24).

Among children and young adolescents, the systematic review and meta-analysis by Antczak and colleagues showed a significant association between vigorous PA and sleep duration (25). Previous research has unveiled positive relationship between objectively assessed PA levels and sleep quality in adolescents (26, 27). PA is often endorsed as a nonpharmacologic approach for improving sleep quality and duration (24, 28, 29). Recent findings among the US adolescent population has revealed positive correlations between sleep duration and PA levels (30). Still, the research literature finds limited support across the lifespan for sex specific associations between sleep and PA (31). PA is reported to be beneficial for some sleep outcomes among adolescents; however, the overall body of evidence is limited, thus warranting more research (32).

While there are established sex differences in sleep duration, sleep quality and PA levels in Norwegian adolescents, it is not yet established if the association between sleep duration and PA levels differ across sex and school levels. Therefore, more research is needed to provide further insight in the association between sleep duration and PA levels. As research evidence points to both social gradients in sleep and PA during adolescence (33, 34), adjusting for socioeconomic status (SES) and age is essential. The aims of this paper were two folded: (1) to describe sleep and PA levels among Norwegian school-based adolescents stratified by school level and sex and (2) to explore the association between sleep and PA.

We hypothesized that adolescents’ sleep duration would be a predictor for higher levels of physical activity among boys and girls in both lower and upper secondary school.

## 2 Methods

This current study has structured the reporting according to the Strengthening the Reporting of Observational Studies in Epidemiology (STROBE) guidelines (35), please see Supplementary file 1.

### 2.1 Data collection

The Ungdata survey is an annual Norwegian survey distributed in schools across the country. Different counties and municipalities are invited each year, and within a three-year period, data have usually been collected from all parts of Norway. Therefore, most annual reports from Ungdata are based upon a representative sample derived from the last 3 years. Reports are formally conducted by Norwegian Social Research (NOVA) at Oslo Metropolitan University in collaboration with the regional center for drug rehabilitation (KoRus) and the municipal sector’s organization (KS). Ungdata comprises both an optional module and a mandatory module. In the optional module, the municipalities are provided with a set of questions possible to incorporate to their survey. The participating Norwegian municipalities provide information to their region and corresponding schools, and the survey is conducted electronically during a school hour. The 2022 Ungdata was primarily conducted in March and April. As participation is voluntary, non-participating adolescents are provided with other schoolwork assigned by their respective teacher. Ungdata is financed from the Norwegian national budget through grants from the Norwegian Directorate of Health (36).

### 2.2 Study design and participants

This study employed cross-sectional data from the 2022 Norwegian Ungdata Survey. The study includes Norwegian adolescents from lower (age 13 to 16 years) and upper secondary school (age 16 to 19 years). As Ungdata is a national survey, all Norwegian adolescents in the respective schools of the included municipalities were invited to participate. As sleep duration was part of the optional module in Ungdata (not in the mandatory module), the question was selected to be incorporated in the following Norwegian counties in lower secondary school: Rogaland, Møre and Romsdal, Nordland, Viken and Agder, and corresponding 40 municipalities. For upper secondary school, the question was incorporated in Møre and Romsdal, Viken and Agder including 28 municipalities, totaling 63,113 responders. The Norwegian Ungdata Surveys tend to have an overall high response rate, which was observed in the study variables of this paper. Please see Supplementary file 2 for more information.

### 2.3 Variables

Ungdata survey entails demographic measures and a broad range of health-related questions. To assure anonymity, grade level is reported as proxy of age. Sex was assessed using three categories’ “Boys,” “Girls” and “Other.”

#### 2.3.1 Exposure

Sleep duration was measured using the question “*How many hours of sleep did you get last night.”* Seven response alternatives were provided, ranging from, 6 h or less or hourly up to 12 h or more. Sleep problems was assessed using the statement “Had sleep problems.” Responders could choose four categories ranging from not at all bothered to a lot bothered. Being tired in school or in leisure activities was measured using four response alternatives, “no days,” “1–2 days,” “3–4 days” and “5 days or more.”

#### 2.3.2 Outcome

Physical activity (PA) levels were assessed using the question, “*How often are you so physically active that you become short of breath or sweaty?*.” Respondents could choose from six response alternatives ranging from “rare” to different times a week, up to “at least 5 times a week.” To assure statistical strength and representativity in regressions, the following categories were recoded into one reference variable: inactivity, rarely and 1–2 times a month. Single-item measures of PA have demonstrated strong reliability and concurrent validity (37). The single-item measure of PA is considered a potentially useful assessment tool for evaluating changes in moderate-vigorous PA levels, especially when device-based measures or longer questionnaires are impractical (38). This PA question was part of the mandatory module of the Ungdata survey, included in all participating municipalities.

#### 2.3.3 Covariates

Relevant covariates in regression models were SES and grade level (age). Ungdata provides a validated construct for SES (39), based on four categories from the Family Affluence Scale II derived from WHO by Currie and colleagues (40, 41). These categories indicate factors such as the parental perception of the family economy, parental educational level and level of prosperity. The measure is presented by using four categories, ranging from 0 to 3, whereas 0 represent the lowest degree of SES and 3 the highest degree of SES.

### 2.4 Ethical consideration

The Norwegian Agency for Shared Services in Education and Research (ref. 821,474), known as SIKT (42), has approved questions used in Ungdata. Participation in the study was voluntary. Informed written consent was obtained from all participants. For younger adolescents, an additional passive approval from parental was given prior to the survey, as the parents received information 2 weeks before the survey and could withdraw their adolescent for participation if preferred. The Norwegian law states that adolescents under the age of 16 years and younger need parental consent on health issues and the passive approval is approved by SIKT for Ungdata. The Ungdata study is conducted in accordance with the Helsinki Declaration.

### 2.5 Statistical analyses

All statistical analyses were performed using IBM SPSS Statistics for Windows, Version 25.0 (IBMCorp., Armonk, NY, United States). Descriptive measures for continuous variables are reported as means and corresponding standard deviation (SD). Categorical variables are presented as counts and percentages. Descriptive measures were stratified by sex and school level. Multinominal logistic regressions were conducted to explore the association between the sleep duration (categorical graded independent variable) and the PA levels (dependent variable). To assure statistical strength and representative baseline for interpreting the effects of the categorical variables and thus enable a clarity of interpretation of lower PA levels compared to the weekly PA levels in the logistic regressions, a recoded reference variable for PA was considered appropriate. Regression models were presented by school level and by sex, stratified using crude analyses and adjusted for SES and grade level. *p*-values <0.05 were considered statistically significant, and all tests were two-sided. Given the high response rate in the presented study variables and large sample size, imputation nor bootstrapping was performed.

## 3 Results

### 3.1 Participants

The total sample included 63,113 adolescents from lower and upper secondary schools across five counties, with the largest representation from Viken (Table 1). The sample was nearly equally distributed between sex. Most adolescent derived from a high SES background (60.4%).

**Table 1:** Sample characteristics (*n* = 63,113).

	N (%)
Sex^a^	
Girls	31,677 (51.5%)
Boys	29,889 (48.5%)
School level	
Lower secondary school	39,110 (62.0%)
Upper secondary school	24,003 (38.0%)
Grade level^b^	
8^th^ grade	13,181 (21.2%)
9^th^ grade	12,383 (19.9%)
10^th^ grade	12,656 (20.4%)
1^st^ grade	9,833 (15.8%)
2^nd^ grade	8,170 (13.1%)
3^rd^ grade	5,946 (9.6%)
County	
Agder	15,240 (24.1%)
More and Romsdal	1,493 (2.4%)
Nordland	1,619 (2.6%)
Rogaland	1,652 (2.6%)
Viken	43,109 (68.3%)
Socioeconomic status^c^	
Low (0–1)	2,293 (3.6%)
Middle (1–2)	22,617 (36.0%)
High (2–3)	37,992 (60.4%)

a *N = 61,566.*

b 62,169.

c 62,902.

### 3.2 Descriptive data of sleep and physical activity levels

In lower secondary school, 57.0% of girls and 44.7% of boys reported sleeping 7 h or less (Table 2). Girls more frequently reported being quite or a lot bothered by sleep problems compared to boys (36.3% vs. 23.1%) and feeling more tired at school or during leisure activities most days (30.2% vs. 18.0%). One out of five girls and one out of three boys reported being active at least 5 days a week.

**Table 2:** Sleep characteristics and physical activity levels for girls and boys in lower secondary school.

	Girls (N %)	Boys (N %)
Sleep duration^a^		
6 h or less	4,944 (25.7%)	3,276 (17.4%)
7 h	6,028 (31.3%)	5,134 (27.3%)
8 h	5,477 (28.4%)	6,165 (32.8%)
9 h	2092 (10.9%)	3,153 (16.8%)
10 h	512 (2.7%)	736 (3.9%)
11 h	115 (0.6%)	170 (0.9%)
12 h or more	87 (0.5%)	160 (0.9%)
Had sleep problems^b^		
Not at all bothered	5,129 (27.0%)	7,371 (40.2%)
Not much bothered	6,971 (36.7%)	6,729 (36.7%)
Quite bothered	4,150 (21.8%)	2,849 (15.5%)
A lot bothered	2,752 (14.5%)	1,398 (7.6%)
Being tired in school or in leisure activities^c^		
No days	6,552 (34.5%)	9,186 (49.8%)
1–2 days	6,679 (35.2%)	5,920 (32.1%)
3–4 days	3,367 (17.7%)	1952 (10.6%)
5 days or more	2,379 (12.5%)	1,370 (7.4%)
Physical activity levels^d^		
Never	348 (1.8%)	326 (1.8%)
Rarely	1,537 (8.1%)	1,001 (5.4%)
1–2 times a month	1,245 (6.5%)	659 (3.6%)
1–2 times a week	5,161 (27.1%)	3,450 (18.6%)
3–4 times a week	6,949 (36.5%)	6,363 (34.3%)
At least 5 times a week	3,785 (19.9%)	6,733 (36.3%)

a *N = 38,049.*

b *N = 37,249.*

c *N = 37,405.*

d *N = 37,557.*

In upper secondary school, 74.9% of girls and 74.3% of boys reported sleeping 7 h or less (Table 3). Girls reported being quite or very bothered by sleep problems more frequently compared to boys (38.7% vs. 30.0%), feeling more tired at school or during leisure activities most days (37.5% vs. 24.8%), and being less physically active at least 5 days a week (17.7% vs. 33.4%).

**Table 3:** Sleep characteristics and physical activity levels for girls and boys in upper secondary school.

	Girls (N %)	Boys (N %)
Sleep duration^a^		
6 h or less	4,934 (39.7%)	4,091 (36.9%)
7 h	4,374 (35.2%)	4,154 (37.4%)
8 h	2,376 (19.1%)	2,140 (19.3%)
9 h	559 (4.5%)	502 (4.5%)
10 h	92 (0.7%)	116 (1.0%)
11 h	37 (0.3%)	31 (0.3%)
12 h or more	50 (0.4%)	61 (0.5%)
Had sleep problems^b^		
Not at all bothered	2,953 (24.1%)	3,726 (34.5%)
Not much bothered	4,554 (37.2%)	3,847 (35.6%)
Quite bothered	2,831 (23.1%)	2089 (19.3%)
A lot bothered	1911 (15.6%)	1,152 (10.7%)
Being tired in school or in leisure activities^c^		
No days	2,855 (23.3%)	4,059 (37.3%)
1–2 days	4,821 (39.3%)	4,121 (37.9%)
3–4 days	2,830 (23.1%)	1704 (15.7%)
5 days or more	1761 (14.4%)	988 (9.1%)
Physical activity levels^d^		
Never	259 (2.1%)	225 (2.1%)
Rarely	1,050 (8.5%)	587 (5.4%)
1–2 times a month	1,168 (9.5%)	571 (5.2%)
1–2 times a week	3,982 (32.4%)	2,405 (22.0%)
3–4 times a week	3,664 (29.8%)	3,485 (31.9%)
At least 5 times a week	2,177 (17.7%)	3,646 (33.4%)

a *N = 23,517.*

b *N = 23,063.*

c *N = 23,139.*

d *N = 23,219.*

### 3.3 Associations between sleep duration and physical activity levels

Crude and adjusted regressions revealed that sleep duration was a significant predictor for being physical active among girls in lower secondary school (all *p* < 0.01; Table 4). A tendency of higher odds progressed by higher PA levels was unveiled. The highest odds were revealed for being active at least 5 times a week (*B* = 1.27; 95% CI [1.21 to 1.32]) and the lowest odds was revealed for being active 1–2 times a week (*B* = 1.07; 95% CI [1.03 to 1.11]).

**Table 4:** Logistic regressions between sleep duration (independent variable) on physical activity levels (dependent variable) for girls in lower secondary school.

	Crude			Adjusted		
	*Exp(B)*	95% CI	*p-value*	*Exp(B)*	95% CI	*p-*value
Reference category						
1–2 times a week	*1.1*	1.05 to 1.14	<0.01	1.07	1.03 to 1.11	<0.01
3–4 times a week	*1.18*	1.14 to 1.23	<0.01	1.14	1.09 to 1.18	<0.01
More than 5 times	*1.31*	1.26 to 1.37	<0.01	1.27	1.21 to 1.32	<0.01

*Adjusted for socioeconomic status and grade level (age).

Regressions revealed that sleep duration was a significant predictor of being physically active 3–4 times a week (*B* = 1.06; 95% CI [1.01 to 1.11]) and at least 5 times a week (*B* = 1.22; 95% CI [1.17 to 1.27]) among boys in lower secondary school after adjusting for SES and grade level (Table 5). Being active 1–2 times a week revealed nonsignificant findings (*B* = 0.99; 95% CI [0.94 to 1.03]).

**Table 5:** Logistic regressions between sleep duration (independent variable) and physical activity levels (dependent variable) for boys in lower secondary school depicted by models 1–3.

	Crude			Adjusted		
	*Exp(B)*	95% CI	*P-value*	*Exp(B)*	95% CI	*p-*value
Reference category						
1–2 times a week	*0.99*	0.94 to 1.04	0.56	0.99	0.94 to 1.03	0.54
3–4 times a week	*1.06*	1.02 to 1.11	<0.01	1.06	1.01 to 1.11	0.01
More than 5 times	*1.2*	1.15 to 1.25	<0.01	1.22	1.17 to 1.27	<0.01

*Adjusted for socioeconomic status and grade level (age).

In upper secondary school, both crude and adjusted regressions revealed that sleep duration was a significant predictor of being physically active among girls (all *p* < 0.01; Table 6). The same trend of higher odds with increased PA levels was observed. After adjusting for SES and grade level (age), the strongest association was found for being active at least 5 times a week (*B* = 1.32; 95% CI [1.24 to 1.40]).

**Table 6:** Logistic regressions between sleep duration (independent variable) on physical activity levels (dependent variable) for girls in upper secondary school depicted by models 1–3.

	Crude			Adjusted		
	*Exp(B)*	95% CI	*p-value*	*Exp(B)*	95% CI	*p-*value
Reference category						
1–2 times a week	*1.09*	1.03 to 1.15	<0.01	1.07	1.01 to 1.13	0.02
3–4 times a week	*1.19*	1.13 to 1.26	<0.01	1.16	1.10 to 1.22	<0.01
More than 5 times	*1.36*	1.29 to 1.45	<0.01	1.32	1.24 to 1.40	<0.01

*Adjusted for socioeconomic status and grade level (age).

In upper secondary schools, crude and adjusted regressions revealed that sleep duration was a significant predictor of being physically active more than 5 times a week (*B* = 1.22; 95% CI [1.15 to 1.30]) after adjusting for covariates (Table 7). However, being active 1–2 times a week (*B* = 0.95; 95% CI [0.89 to 1.02]) and 3–4 times a week (*B* = 1.01; 95% CI [0.95 to 1.08]) showed nonsignificant associations.

**Table 7:** Logistic regressions between sleep duration (independent variable) on physical activity levels (dependent variable) for boys in upper secondary school depicted by models 1–3.

	Crude			Adjusted		
	*Exp(B)*	95% CI	*p-value*	*Exp(B)*	95% CI	*p-*value
Reference category						
1–2 times a week	*0.97*	0.91 to 1.05	*0.45*	0.95	0.89 to 1.02	0.19
3–4 times a week	*1.04*	0.98 to 1.12	*0.21*	1.01	0.95 to 1.08	0.76
More than 5 times	*1.26*	1.19 to 1.35	<0.01	1.22	1.15 to 1.30	<0.01

*Adjusted for socioeconomic status and grade level (age).

## 4 Discussion

In this study, we aimed to describe sleep and PA levels, and to explore the association between sleep and PA among Norwegian school-based adolescents, stratified by school level and sex. The majority of adolescents in upper secondary school did not achieve the recommended 8 h of sleep, and girls in lower secondary school reported shorter sleep durations than boys. Across school levels, girls reported higher levels of sedentary behavior and were less engaged in PA than boys. Sleep duration was a predictor of being active at least 5 times per week across both sexes and school levels, with increased odds ranging from 22 to 32%.

Overall, the current results confirm that a significant proportion of adolescents, about half of those in lower secondary and a majority in upper secondary did not achieve the recommended amount of sleep (4). Our finding that 3 out of 4 upper secondary students do not adhere to sleep recommendations, with more girls reporting sleep problems, aligns with previous studies (6, 7). The rate of non-compliance with sleep recommendations in upper secondary school is somewhat lower than that reported by Saxvig and colleagues (6). This discrepancy may be due to differences in the methods used to assess sleep duration. For example, Saxvig et al. employed a more comprehensive approach, estimating sleep duration through a two-step process that included both bedtime to shuteye time and shuteye time to sleep onset (6), potentially yielding more precise estimates. Nonetheless, the larger sample from the Ungdata study complements previous research by offering a nationwide perspective across school levels.

Interestingly, girls in lower secondary school were more likely than boys to fall short of the 8-h sleep recommendation. This contrasts with other studies of younger and older adolescents, which often report similar sleep durations across sexes, with boys sometimes showing slightly shorter sleep durations (6, 7, 43, 44). For instance, findings among Australian adolescents indicated no significant sex differences in objectively measured sleep parameters (45). However, most studies tend to report shorter sleep durations among boys (46–50), although exceptions exist (51). It is also worth noting that boys may overestimate self-reported sleep duration more than girls when compared with actigraphy data (52). Another possible explanation for our findings could be that younger girls perceived the final period of Covid-19 restrictions differently than boys, as girls have reported struggling more with mental health during this time (53, 54). Although this study was conducted after most Covid-19 restrictions were lifted, secondary schools may still have had some restrictions in place, such as prolonged digital teaching and less in-person instruction compared to lower secondary schools. Additionally, because the Ungdata survey was administered on different days of the week and the sleep question only assessed sleep the previous night, there may have been differences in sleep patterns on Mondays compared to other days, as younger Norwegian girls have previously reported longer sleep durations on weekends than boys (43).

Given the consistently higher proportion of sleep problems and lower physical activity (PA) levels in girls, it is possible that various coinciding factors are at play. Previous research suggests that moderate to vigorous PA improves sleep quality among adolescents (26, 27, 55). In support of this, Fonseca et al. reported in their review that longer sleep durations and better sleep quality are associated with higher levels of physical fitness in adolescents (56). The PA levels observed in our study align with previously reported trends for this population (15), highlighting a concerning pattern: only half as many Norwegian girls report high levels of PA compared to boys.

As hypothesized, sleep duration showed the strongest association with the highest PA levels across both sex and school levels, which is consistent with previous research (25–27, 32, 55, 56). Several mechanisms may explain these associations, as longer sleep facilitates key prerequisites for being physically active. Adequate sleep promotes optimal recovery and energy restoration in adolescents, presumably reducing fatigue and increasing motivation and self-efficacy (43, 57). Higher energy levels, coupled with increased motivation and belief in one’s abilities, are fundamental for engaging in PA, particularly at higher intensity levels. Additionally, consistent sleep routines and structures are likely crucial factors that support the implementation of PA, and there may be reciprocal associations at work. We assume that these factors are mutually reinforcing during adolescence: sleep duration facilitates higher PA levels, and PA, in turn, promotes better and longer sleep. This aligns with previous suggestions of a bidirectional relationship between sleep and PA (24).

According to an umbrella review on PA and sleep across the lifespan, there was limited evidence supporting sex-specific associations (31). However, our regressions revealed notable sex-specific differences, with sleep duration emerging as a strong predictor for all weekly PA levels among girls, across school levels. Adolescence is a complex developmental phase, during which girls undergo different hormonal changes than boys. Sex-specific hormones such as estradiol and follicle-stimulating hormone (FSH) have been linked to longer sleep duration and poorer sleep quality (58). Studies among junior athletes have shown sex differences in both subjective and objective sleep parameters in relation to the menstrual cycle (59, 60), aligning with reports of increased fatigue and iron deficiency among girls due to menstruation (61, 62), which likely impacts their PA levels. Another potential explanation could be that girls typically enter puberty and experience growth spurts earlier than boys, which might necessitate more recovery at an earlier stage.

Finally, while longer sleep is associated with higher PA levels, it is important to consider the nuances. A linear relationship between sleep duration and PA levels may not always be present, as underlying pathological conditions could affect daytime capacity in children and adolescents who require more than 12 h of sleep. This was highlighted by Kobel and colleagues, who found that children in the highest sleep category showed a negative association with meeting PA guidelines (63). Nevertheless, given that most Norwegian adolescents do not meet sleep recommendations, longer sleep remains a favorable prerequisite for higher levels of PA.

## 5 Strength and limitations

A major strength of this study lies in the large sample of Norwegian adolescents drawn from various regions across the country, combined with a high response rate across all included study variables. This enhances the validity, representativity and generalizability of the findings. Additionally, the Ungdata survey employs a validated measure of SES and, since 2010, has developed rigorous methods for cleaning raw data, such as identifying unreliable or insincere responses (e.g., maximum scores in both depression and life satisfaction measures) (39). However, as much of the study sample comes from higher SES backgrounds, our findings may not be generalizable to other populations. Other limitations should also be considered, such as the non-validated single-item questions from Ungdata. Exploring other potential directions in the associations between sleep duration and physical activity could have provided additional insights. The cross-sectional design of the study does not allow for causal interpretations and offers only a snapshot of the current situation without a temporal component.

Furthermore, we chose to recode three categories into one physical activity reference category to ensure sufficient statistical power, a more representative baseline, and clearer interpretations. However, this recoding reduces variability in the estimates, which could be seen as a limitation. Additionally, the assessment of sleep duration relies on a single item in which adolescents report their sleep duration using predefined response categories. The broad nature of these categories may yield less nuanced results compared to more specific sleep measures. Future studies should consider including more detailed assessments of sleep duration, such as sleep onset latency, wake after sleep onset, and specific bedtime and wake time. In addition, future research should explore the causes of adolescents not responding to questions of sleep and PA.

## 6 Implications

Adolescence is a critical period of transition from childhood to adulthood, during which the establishment of lifelong healthy habits is essential. Adolescents, along with their support networks—such as school administrators, teachers, trainers, and parents—require up-to-date, research-based evidence to make informed and healthy decisions. Our study adds to the existing body of research by showing that more Norwegian girls than boys report getting less sleep than recommended in lower secondary school. Additionally, sleep duration is a predictor of high PA levels during adolescence, across school levels and sex, even after adjusting for SES and age. These findings highlight short sleep duration and low PA levels as significant public health concerns among adolescents, with girls being particularly affected. Policies and practices should empower adolescents to make informed health choices by offering clearer recommendations and guidelines through the interdisciplinary subject “Public Health and Life Skills” in both lower and upper secondary school, as enhancing health literacy may positively impact both PA levels and sleep (64, 65).

## 7 Conclusion

This study reveals that approximately half of younger adolescents and three-quarters of older adolescents do not meet the recommended minimum of 8 h of sleep per night. In lower secondary school, more girls than boys reported shorter sleep durations, and across all school levels, girls consistently reported more sleep problems and lower levels of PA than boys. Furthermore, sleep duration was consistently associated with a 20 to 30% higher likelihood of being active at least 5 days a week, regardless of sex or school level. These findings highlight the crucial role of adequate sleep in promoting higher PA levels among Norwegian adolescents. Consequently, the Norwegian government and policymakers should promote healthy sleep habits by explicitly incorporating sleep guidelines into the school curriculum to support increased PA levels.

## Data Availability

The datasets that support the findings of this study are available upon reasonable request from the Norwegian Agency for Shared Services in Education and Research (SIKT) (42). Requests to access these datasets should be directed to https://sikt.no/.
