# Supplementary material for: The relationship between sleep duration and physical activity level among Norwegian adolescents: a cross-sectional study
Source: Front Public Health. 2024 Nov 28;12:1495826. doi: 10.3389/fpubh.2024.1495826 (PMC11634845; doi:10.3389/fpubh.2024.1495826)
Supplement: Supplementary file 2 [file Table_2.docx]

Supplementary File 2

**Supplementary table** Response rate in study variables of the total sample N=63,113

| Variable | N | Response rate % |
| --- | --- | --- |
| Sex  School level  Grade level  County  SES  Sleep duration | 61566  63113  62169  63113  62902  61766 | 97.5%  100%  98.5%  100%  99.7%  97.9% |
| Had sleep problems  Being tired in school or in leisure activities  PA | 60312  60544  60776 | 95.6%  95.9%  96.3% |
